# Supplementary material for: Magnetic resonance imaging analysis predicts nanoparticle concentration delivered to the brain parenchyma
Source: Commun Biol. 2022 Sep 15;5:964. doi: 10.1038/s42003-022-03881-0 (PMC9477799; doi:10.1038/s42003-022-03881-0)
Supplement: Supplementary file 2 — Supplementary Information [file 42003_2022_3881_MOESM2_ESM.pdf]

Magnetic Resonance Imaging Analysis Predicts Nanoparticle Concentration Delivered to the Brain Parenchyma

Michael Plaksin[1*¥], Tiran Berkovitz[1*], Gabriella Toltsis[1], Javier Grinfeld[1], Boaz Shapira[1], Yuval Zur[1], Rafi de Picciotto[1], Eyal Zadicario[1], Mustaffa Siddeeq[2], Anton Wohl[2], Zion Zibly[2], Yoav Levy[1], Zvi R. Cohen[2¥]

[1]Insightec Company Ltd., Tirat Carmel 39120, Israel

[2]Neuro Oncology Unit, Sheba Medical Center, Ramat Gan 52621, Israel

affiliated with the Sackler School of Medicine Tel Aviv University, Israel

* Equal contributors

¥ Corresponding authors: MichaelP@insightec.com
Zvi.Cohen@sheba.health.gov.il

Supplementary Information

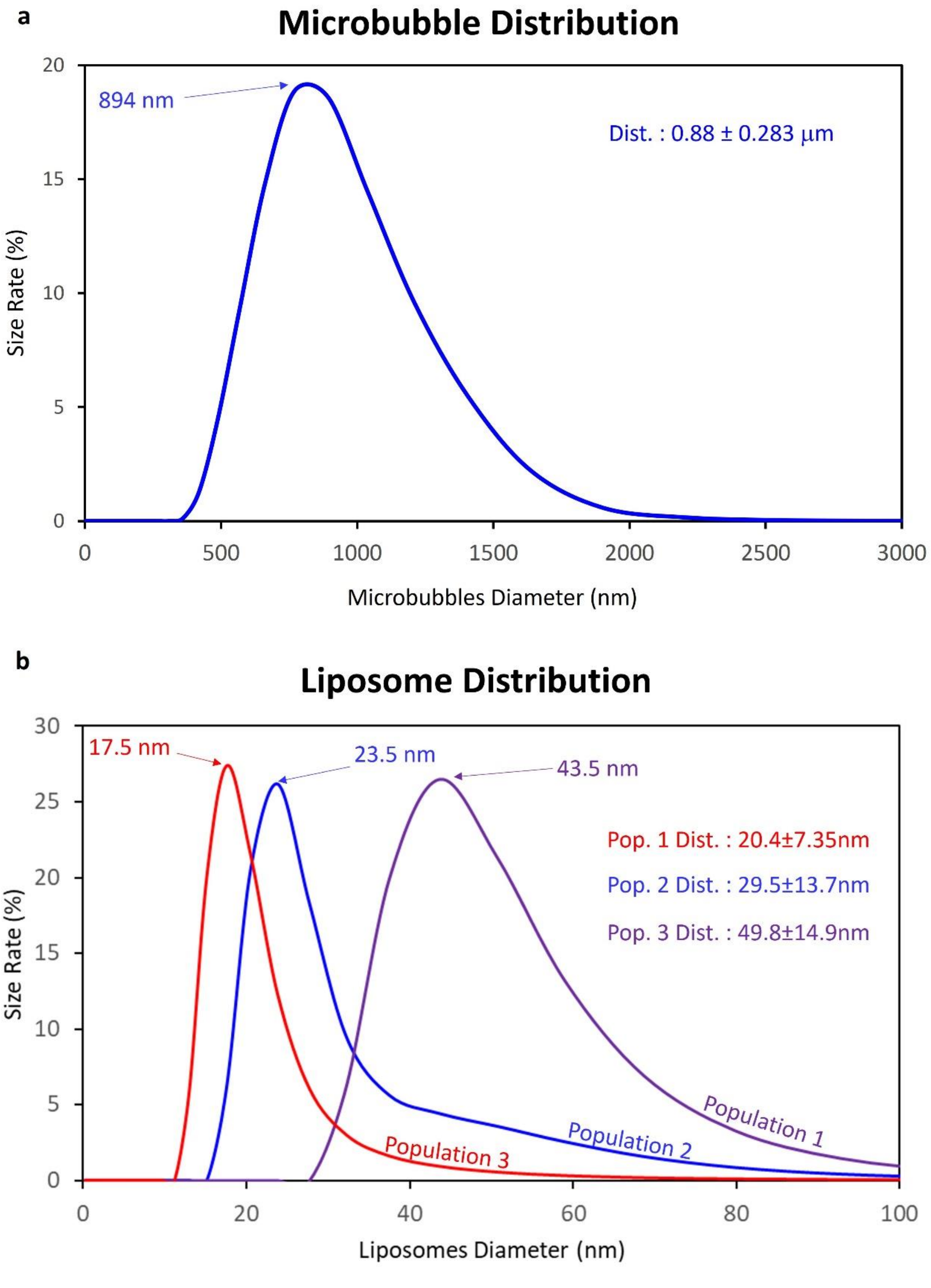

Supplementary Fig. 1 Particle Distribution. Microbubbles (**a**) and Gd-liposome population (**b**) distributions. Numbers above the curves indicate the distribution peaks. The mean and standard deviation of each population are indicated on the right side of the figures.

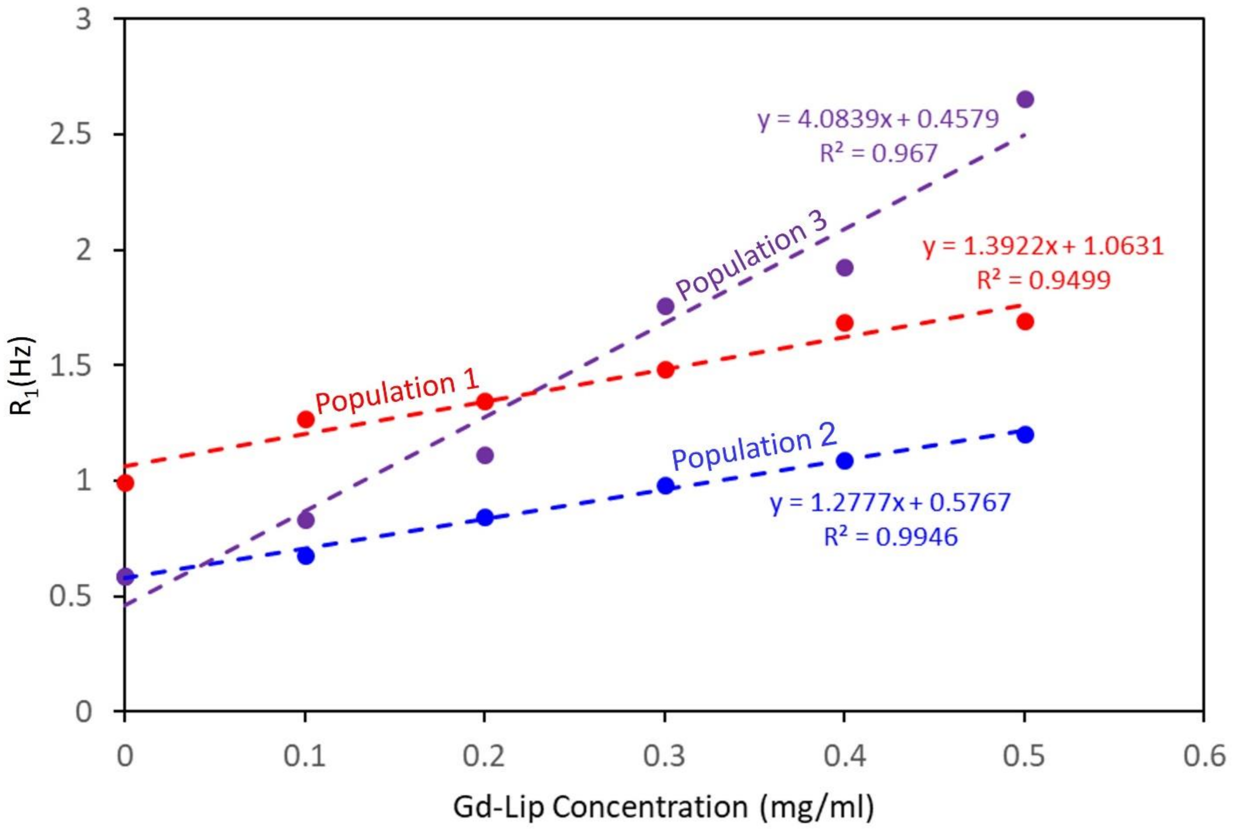

Supplementary Fig. 2 R_1 to Gd-liposome concentration calibration. The calibration was performed using polycarbonate well arrays, each containing 0.5 ml rat blood with different Gd-liposome concentrations. The rat blood content inside each liposome's population measurement remained the same, to keep the same R_1 baseline. Each calibration was done using the blood from a single rat.

Population 1 - Gd liposomes 18 nm, Population 2 - Gd liposomes 24 nm, and Population 3 - Gd liposomes 44 nm.

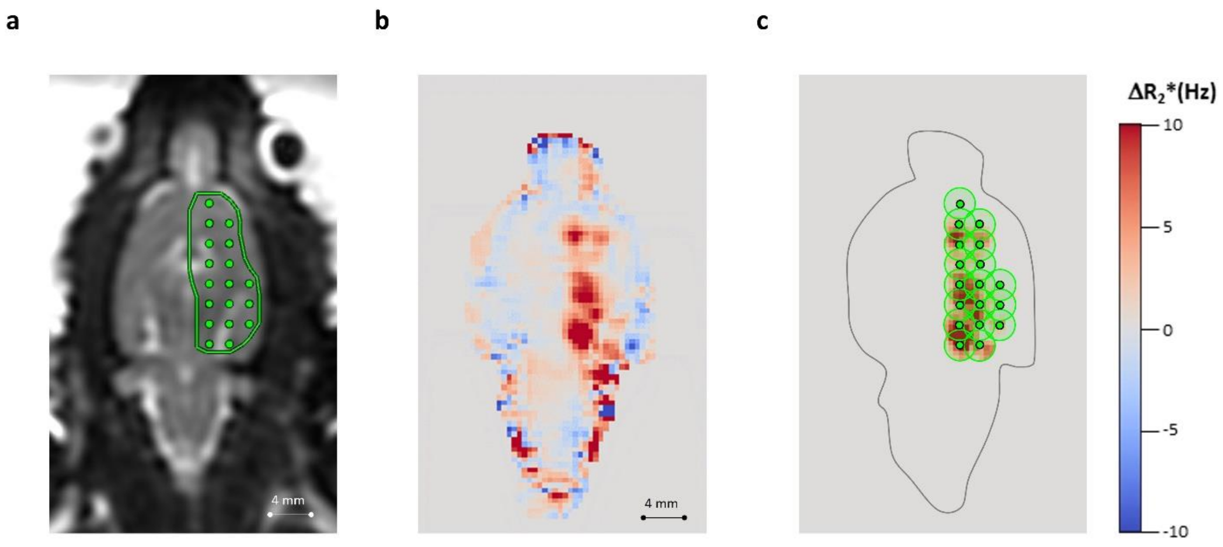

Supplementary Fig. 3 ΔR_2* map and its relation to the sub-spot locations. a Sub-spot locations as defined in the treatment T_2-weighted planning images. **b** ΔR_2* map and the relationship between its values in the treatment area and the points where the ultrasound was applied (**c**); the diameter of the green circles is the lateral spot width (3 mm) of the ultrasound beam.

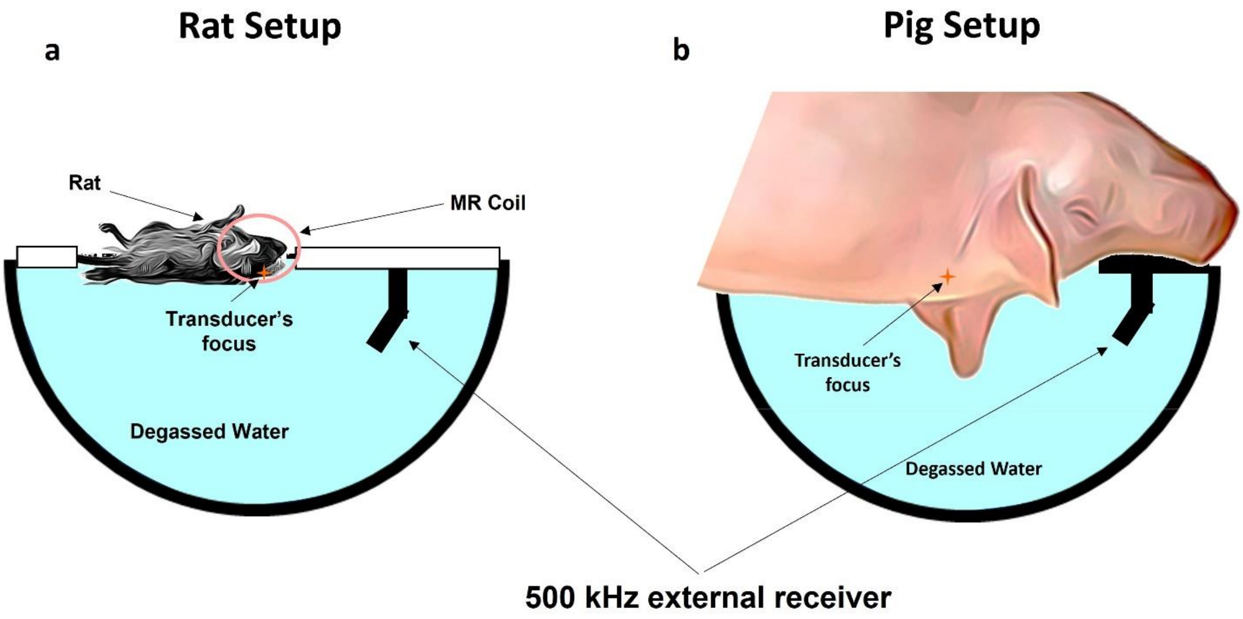

Supplementary Fig. 4 Power spectrum measurement setups. Rat Setup (**a**) and Pig Setup (**b**). The US transducer and the external US receiver were the same for both the rat and pig experiments.
