# Supplementary material for: Magnetic resonance imaging analysis predicts nanoparticle concentration delivered to the brain parenchyma
Source: Commun Biol. 2022 Sep 15;5:964. doi: 10.1038/s42003-022-03881-0 (PMC9477799; doi:10.1038/s42003-022-03881-0)
Supplement: Supplementary file 3 — Description of Additional Supplementary Files [file 42003_2022_3881_MOESM3_ESM.pdf]

# Description of Additional Supplementary Files

**File name:** Supplementary Data 1
**Description:** The source data for plots in Figure 5.
